# CADA—computer-aided DaTSCAN analysis

**DOI:** 10.1186/s40658-016-0140-9

**Published:** 2016-02-16

**Authors:** Antonio Augimeri, Andrea Cherubini, Giuseppe Lucio Cascini, Domenico Galea, Maria Eugenia Caligiuri, Gaetano Barbagallo, Gennarina Arabia, Aldo Quattrone

**Affiliations:** Institute of Molecular Bioimaging and Physiology of the National Research Council (IBFM-CNR), Catanzaro, Italy; Institute of Radiology, Nuclear Medicine Unit, University Magna Graecia, Catanzaro, Italy; Institute of Neurology, University Magna Graecia, Catanzaro, Italy

**Keywords:** Parkinson’s disease, DaTSCAN, Computer-aided diagnosis

## Abstract

**Background:**

Dopamine transporter (DaT) imaging (DaTSCAN) is useful for the differential diagnosis of parkinsonian syndromes. Visual evaluation of DaTSCAN images represents the generally accepted diagnostic method, but it is strongly dependent on the observer’s experience and shows inter- and intra-observer variability. A reliable and automatic method for DaTSCAN evaluation can provide objective quantification; it is desirable for longitudinal studies, and it allows for a better follow-up control. Moreover, it is crucial for an automated method to produce coherent measures related to the severity of motor symptoms.

**Methods:**

In this work, we propose a novel fully automated technique for DaTSCAN analysis that generates quantitative measures based on striatal intensity, shape, symmetry and extent. We tested these measures using a support vector machine (SVM) classifier.

**Results:**

The proposed measures reached 100 % accuracy in distinguishing between patients with Parkinson’s disease (PD) and control subjects. We also demonstrate the existence of a linear relationship and an exponential trend between pooled structural and functional striatal characteristics and the Unified Parkinson’s disease Rating Scale (UPDRS) motor score.

**Conclusions:**

We present a novel, highly reproducible, user-independent technique for DaTSCAN analysis producing quantitative measures directly connected to striatum uptake and shape. In our method, no a priori assumption is required on the spatial conformation and localization of striatum, and both uptake and symmetry contribute to the index quantification. These measures can reliably support a computer-assisted decision system.

**Electronic supplementary material:**

The online version of this article (doi:10.1186/s40658-016-0140-9) contains supplementary material, which is available to authorized users.

## Background

Parkinsonian syndromes include neurological disorders characterized by common clinical features such as rigidity, bradykinesia, resting tremor and postural instability [1]. The most frequent cause of parkinsonian symptoms is Parkinson’s disease (PD), a degenerative pathology of the central nervous system due to progressive degeneration of dopaminergic neurons in the substantia nigra, which in turn results in a loss of dopamine transporters (DaT) in the striatum [2–4].

Imaging the integrity of the nigrostriatal dopaminergic pathway can improve the accuracy of diagnosis in movement disorders and optimize the therapeutic approach [5, 6], especially when dealing with parkinsonian syndromes that respond differently to medications.

Single-photon emission computed tomography (SPECT) provides a valuable tool for dopamine neurotransmission visualization both at the pre-synaptic and post-synaptic levels by means of several tracers [7, 8]. In particular, SPECT imaging with ^123^I-ioflupane (^123^I-FP-CIT, DaTSCAN, GE Healthcare) provides an accurate in vivo marker of the nigrostriatal dopamine terminals, and it is currently one of the most used DaT probes [9–11]. ^123^I-FP-CIT is a pre-synaptic radiopharmaceutical of the DaT presenting a significant uptake decrease in basal ganglia of PD subjects [9] and the most widely used tracer for pre-synaptic evaluations in both Europe and the USA [12]. The utility of this tracer for pre-synaptic evaluations has also been shown in the differential diagnosis between essential tremor (ET) and idiopathic parkinsonism [13–15].

Visual interpretation of DaTSCAN images according to available qualitative assessment criteria [10] has traditionally been used for the diagnosis of parkinsonian symptoms. However, this approach is rater-dependent and prone to errors, since it relies on gross changes in striatal DaT density. For this reason, several methods for quantitative or semi-quantitative DaTSCAN evaluation have been proposed as a valuable tool for obtaining reproducible diagnostic outcomes and for follow-up in longitudinal studies [10, 16].

Semi-quantitative measures for striatal tracer uptake quantification are defined as ratios between the striatal radiopharmaceutical-specific uptake and the non-specific uptake in an area of low DaT density, usually the occipital cortex. The calculation of such quantities requires manual positioning of regions of interest (ROIs) on the SPECT image by a highly experienced operator, which makes the procedure time-consuming and hampered by a considerable intra- and inter-operator variability [17, 18]. In order to reduce analysis time, pre-defined ROIs are also used; but this approach can also be highly arbitrary, especially in case of low striatal uptake [10, 19]. To face this issue, free and commercial software packages [20, 21] have been developed that provide automatic and semi-automatic placing of ROIs. The Brain Registration & Analysis Software Suite (BRASS, Hermes Medical, SE), can automatically quantify tracer uptake in the striatum of each hemisphere and in different subregions [22]. BRASS fits the patient data onto a template containing a set of pre-defined ROIs and calculates the specific binding ratio (SBR) in the whole striatum, the caudate nucleus and the putamen. A very similar approach is used in two other commercial software packages: EXINI dat (EXINI Diagnostic, SE) and DaTQUANT (GE Healthcare). All these commercial products also return information about binding in striatal subregions deriving the caudate-to-putamen uptake ratio that can evaluate the amount of degeneration in the early stage of disease. These methods have shown similar accuracy when compared to manual delineation [23] but often consider only a few 2D slices for uptake quantification and do not provide measurements directly based on the shape of the uptake regions.

In the majority of these studies, the focus is on the use of DaTSCAN to separate different classes of patients or patients from healthy controls. Recently, authors [24] developed and evaluated Spectalizer, a fully automated method for 3D ROI extraction and SBR evaluation. The method performed well in separating patients with PD from those with ET but did not consider shape nor extension features of the striatum. More recent fully automated approaches [25–27], instead, not only segmented striatal ROIs with a thresholding approach, but further reduced the number of voxels to include in the analysis by using a combination of statistical tests and data reduction techniques. Subsequently, the reduced set of voxels was fed into a supervised classifier. None of these methods introduced shape features in the analysis pipeline.

Indeed, shape and asymmetry information about the basal ganglia can be useful not only in PD diagnosis, where striatal damage at the onset presents unilaterally and then spreads to the contralateral structure, but also in differential diagnosis between PD and other parkinsonian syndromes, in which damage could be present in specific spatial patterns.

Furthermore, only one study [20] has assessed the correlation between automatically extracted imaging measures and symptom severity. The lack of this kind of information represents a limitation when validating a diagnostic index, since the presence of a relationship between striatal uptake and clinical symptoms has been demonstrated by several authors [28, 29] and is crucial for the development of an objective marker of disease severity to help understand and monitor disease onset, severity and progression.

For all these reasons, we propose a novel fully automated technique to analyse DaTSPECT images in a 3D fashion. The aim is twofold: (1) to extract relevant quantitative measures and features based on striatal intensity, shape and symmetry and (2) to demonstrate that the use of multimodal information on structural and functional integrity of the striatum may lead to high correlation with clinical measures, thus representing a robust marker of disease progression. To test the discriminating power of selected measures, we built a support vector machine (SVM) classifier and evaluated its accuracy in distinguishing patients with PD from healthy subjects. We also compared the performance of our method with standard qualitative and semi-quantitative assessments.

## Methods

Thirty-one patients with a diagnosis of probable PD (Group I, 18 M/13 F) and 12 age- and sex-matched healthy controls (Group II, 5 M/7 F) underwent DaTSCAN imaging. Demographic and clinical characteristics of the patients and controls are listed in Table 1. Patients with PD diagnosis fulfilled the UK Parkinson’s Disease Society Brain Bank criteria [30], and their clinical diagnoses were confirmed 2 years after the SPECT data acquisition. An expert with years of experience in movement disorders performed neurological examinations and clinical assessments on all patients using the Unified Parkinson’s Disease Rating Scale Motor Examination (UPDRS-ME) [31] and the Hoehn and Yahr (H&Y) rating scale [32] in an off phase (off medications overnight). No patient had any history of other neurological diseases other than PD. Structural magnetic resonance imaging (MRI) excluded the presence of vascular lesions, brain tumours or other abnormalities. The Institutional Ethics Committee of Magna Graecia University (Catanzaro, Italy) approved the study, and all subjects gave written informed consent.

**Table 1:** Demographic and clinical features in PD and HC participants

Characteristic	PD (*n* = 31)	HC (*n* = 12)	*p* value
Age (mean ± SD)	63.77 ± 9.65	62.67 ± 11	NS^a^
F/M	13/18	7/5	NS^b^
Age at onset (mean ± SD)	60.2 ± 6.4	-	-
Duration of disease (mean ± SD)	7.5 ± 5.3	-	-
H&Y (mean ± SD)	2.5 ± 0.5	-	-
UPDRS-ME score (mean ± SD)	29.13 ± 8.65	-	-

*SD* standard deviation, *NS* non-significant, *UPDRS-ME* Unified Parkinson’s Disease Rating Scale Motor Examination

^a^This *p* value was determined using an unpaired *t* test

^b^This *p* value was determined using *χ* ^2^ test

Brain imaging was performed 3 h after the administration of 200 MBq of ^123^I-FP-CIT (GE-Amersham, Eindhoven, The Netherlands) using a dual-headed gamma camera (Infinia Hawkeye, General Electric, Milwaukee, WI) equipped with a low-energy, high-resolution collimator (SPECT). Scans were acquired with a photopeak window centred around 159 KeV ± 10 % with a 128 × 128 image matrix (zoom factor, 1.5, 40 s per view and 2 × 64 views). The slice thickness was 2.95 mm. Images were reconstructed using a Butterworth filter (with a cut-off of 0.5 and an order of 6). Chang’s correction method was used to compensate for attenuation using a coefficient, *μ*, of 0.11 cm^−1^.

Three different experts with several years of experience diagnosing movement disorders according to the criteria described in [10] carried out the qualitative visual assessment of the DaTSCAN images. Semi-quantitative analysis was performed selecting three consecutive slices with the highest striatal uptakes. ROIs with fixed sizes were manually positioned over the left and right striatum, and a third ROI in the occipital cortex was used as reference region. Left and right striatal uptake was quantified separately. For each striatum, specific binding ratio (SBR) was calculated as follows:

1 $$ {\mathrm{SBR}}_{\mathrm{Str}}=\frac{C_{\mathrm{Str}}-{C}_{\mathrm{Occ}}}{C_{\mathrm{Occ}}}, $$

where *C*_Str_ and *C*_Occ_ are, respectively, the mean count of voxels in the striatal ROI and the mean count of voxels in the occipital ROI. Nuclear medicine experts blinded to the clinical data performed the procedure.

**Fig. 1:**
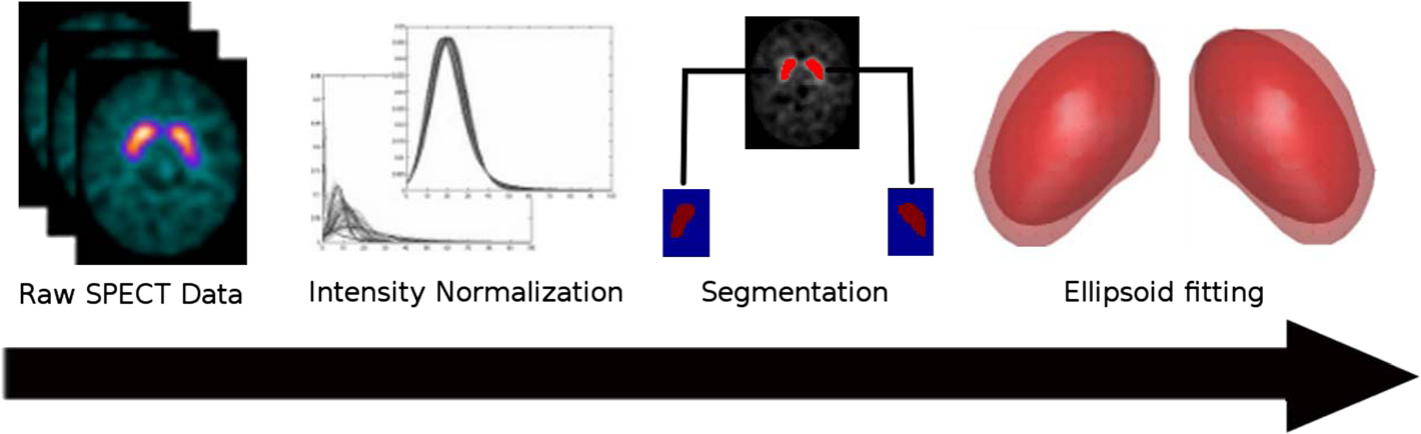
CADA workflow process

The automatic analysis of SPECT volumes consisted of five main steps. Figure 1 shows the entire process. First, we spatially registered and intensity normalized the raw images. In the second phase, we employed a segmentation routine to extract two binary masks corresponding to the left and right striata. Subsequently, we fitted an ellipsoid surface on each mask using a non-linear optimization algorithm. We then used the ellipsoid space to extract a set of features characterizing the basal ganglia. Finally, we put the resulting parameters into a SVM classifier. All stages are discussed in detail in the following subsections.

### Pre-processing

The pre-processing procedure involved two steps. First, we spatially normalized the SPECT images using the FSL-FLIRT tool (Functional Magnetic Resonance Imaging Software Library, Oxford University, UK [33, 34]) in order to ensure that the same voxel refers to the same anatomical position across different volumes. We registered each image to an average image of the control subjects’ group. Since our method does not require a perfect voxel-by-voxel correspondence, but only an overall common position of each subject, we used a six-degrees-of-freedom linear affine transform that prevents deformation of the images. During this step, we obtained a template image (T) by averaging all the co-registered images from the control group. To get a symmetric template, we averaged T with its hemisphere mid-plane reflection. To allow for appropriate comparison of uptake values across subjects, we performed a second pre-processing step in which the images were intensity normalized. We employed linear intensity normalization according to the method described in [35]. The intensity histogram in a SPECT brain image can be fit using a positively skewed *α*-stable distribution. Such a distribution is a generalized case of Gaussian distribution and can be controlled by four parameters: *α*, *β*, *γ* and *μ*, which, respectively, describe impulsiveness, skewness, concentration of samples along the bulk (dispersion) and distribution shift on the *x*-axis (position). By calculating the *α*-stable parameters for each image histogram *i*, it is possible to adjust all dispersions *γ*_i_ and location *μ*_i_ so that they have similar values. This is done by calculating γ* and μ* as the mean values of the *γ*_i_ and *μ*_i_ parameters and by using the following normalization equation:

2 $$ Y={a}_iX-{b}_i, $$

where $$ a=\frac{\gamma }{\gamma_i} $$, and $$ b=\mu -\frac{\gamma^{*}}{\gamma_i}\mu . $$

### Segmentation

We employed a segmentation method based upon a Gaussian mixture model (GMM) to identify regions of high uptake on each subject [36]. The application of GMM relies on the assumption that the intensity value at each voxel in an observed image is sampled from a Gaussian mixture distribution. We assume that all voxels in the image belong to either a high uptake region (striatum) or a low uptake region. In the model, we describe each voxel as a *d*-dimensional (*d* = 4) data vector *x* containing its intensity value and spatial coordinates. The resulting GMM is a weighted sum of two Gaussian densities given by the equation

3 $$ p\left(x\Big|\lambda \right)={\displaystyle {\sum}_{\mathit{\mathsf{i}}=\mathsf{1}}^{\mathit{\mathsf{M}}}{\omega}_iN}\left(x\Big|{\mu}_i,{\varSigma}_i\right), $$

where *ω*_*i*_, *i* = 1,…, *M* are the mixture weights and *N*(*x*|*μ*_*i*_, *Σ*_*i*_), *i* = 1,…, *M* are the Gaussian component densities. Each component density is a four-variate Gaussian function of the form,

4 $$ N\left(x\Big|{\mu}_i,{\varSigma}_i\right)=\frac{1}{{\left(2\pi \right)}^{d/2}{\left|{\varSigma}_i\right|}^{1/2}} \exp \left(-\frac{1}{2}\left(x-{\mu}_i\right)\hbox{'}{\varSigma}^{-1}\left(x-{\mu}_i\right)\right), $$

with mean vector *μ*_*i*_ and covariance matrix *Σ*_*i*_. The mixture weights satisfy the constraint ${\sum}_{\mathsf{i}=\mathsf{1}}^{\mathsf{M}}{\omega}_i=1$. The mean vectors, covariance matrices and mixture weights from all component densities parameterized the complete model. These parameters are collectively represented by the notation

5 $$ \lambda =\left\{{\omega}_i,{\mu}_i,{\varSigma}_i\right\},i=1,\dots, M $$

The expectation-maximization method estimates the mixture parameters [37]. In our implementation, we assumed two Gaussian components. Once the expectation-maximization method has converged, a complete set of parameters are returned and used to compose two distribution functions. These functions are considered probabilistic approximations of how the voxels are partitioned in the image. Thus, the function corresponding to the highest uptake value will better describe the voxels in the striatum. Formally, we carried out the segmentation by assigning each voxel to the proper cluster according to principle of the maximum-likelihood estimation. The *j*th element is labelled *L*_*j*_ according to the following equation

6 $$ {L}_j=\underset{i}{ \max}\frac{ \exp \left(-\frac{1}{2}\left({x}_j-{\mu}_{{}_i}^{*}\right){\left({\varSigma}_{{}_i}^{*}\right)}^{-1}\left({x}_j-{\mu}_{{}_i}^{*}\right)\hbox{'}\right)}{{\left|{\varSigma}_{{}_i}^{*}\right|}^{1/2}}, $$

where *x* is the input data and μ* and Σ* are the two estimated mixture parameters. Since the segmentation method is completely unsupervised and not input-dependent, the two resulting distributions will be slightly different from subject to subject in terms of count rate, thus avoiding the need to specify a priori information or threshold values that could be subject-specific or scanner-specific.

### Ellipsoid fitting

The segmentation step produces a 3D binary mask per side of the brain; each mask delimits a region of high uptake and can extract quantitative measures in terms of peak and mean uptake but is not suitable to determine spatial features such as shape, orientation and position of each striatum. To evaluate striatal spatial features in a reproducible manner, we approximated the set of data points in each mask to an ellipsoid and then derived the metrics of interest from the fit. The fitting procedure minimizes the sum of squares of the distances between the data points and the ellipsoid surface by solving a non-linear optimization problem.

Formally, given a set of points,

$$ {\left\{{X}_i\right\}}_{i=1}^m,m>3,{\left(X-U\right)}^T{R}^TD\ R\left(X-U\right)=1, $$

where *U* is the ellipsoid centre, *R* is an orthonormal matrix representing the ellipsoid orientation and *D* is a diagonal matrix whose diagonal entries represent the reciprocal of the squares of the half-lengths of the ellipsoid axes; the fit is obtained by minimizing the following energy function:

7 $$ E\left(U,R,D\right)={\sum}_{\mathsf{i}=\mathsf{1}}^{\mathsf{m}}{\left({L}_i-r\right)}^2, $$

where *L*_*i*_ is the distance from the point *X*_*i*_ to the ellipsoid.

### Features extraction

For each subject, we calculated two measures per side: the mean ellipsoid uptake (MEU) and a dysmorphic index (DI). We calculated the MEU by averaging the uptake values in a ROI defined by the fitted ellipsoid, which can be interpreted in the same way as the SBR in the semi-quantitative approach; the larger the MEU, the higher the uptake. We instead obtained the DI by comparing the orientation of each striatum with the orientation of the corresponding striatum segmented from the average template image (See Additional file 1). To do this, instead of comparing the nuclei directly, we use the relative ellipsoids. The fitted ellipsoid is a good approximation of the striatum. When the shape of the striatum changes as result of the diseases, it has an impact on the ellipsoid and its orientation. The ellipsoid orientation is defined through the eigenvectors; thus, the problem of comparing two ellipsoids can be solved by comparing the corresponding eigenvectors. By calculating the absolute value of the dot product between each eigenvector from the template and the corresponding eigenvector from the subject’s striatum, we obtained a tern of values included in the interval (0, 1). Each value in the tern is a measure of the nucleus alignment in the corresponding direction. Finally, we calculated DI by multiplying all the values in the tern and subtracting the result from one as follows:

8 $$ \mathrm{D}\mathrm{I}=1-{\displaystyle {\prod}_{\mathit{\mathsf{i}}=\mathsf{1}}^{\mathsf{3}}}\left|\left({E}_i^S\cdot {E}_i^T\right)\right| $$

where *E*_*i*_^*S*^ and *E*_*i*_^*T*^ are the *i*th ellipsoid subject eigenvector and the *i*th ellipsoid template eigenvector, respectively.

When the striatum has the characteristic “comma-shape”, the ellipsoid fitted on it retains a very similar orientation to the template ellipsoid. All the quantities in the tern will be very close to one and then DI will be close to zero, indicating a non-significant change in the striatum shape. On the contrary, when the ellipsoid is fitted on a damaged nucleus, it is expected to be warped and with a different orientation with respect to the template. So one or more values in the tern will be close to zero and DI will be close to one, indicating a high degree of dysmorphism.

### Classification

To investigate the discriminating power of the extracted measures, we built different SVM classifiers to distinguish PD patients from control subjects and trained each classifier using a different dataset of measures. Overall, we tested four configurations:

- Experiment I: the feature vector is composed by left and right MEU values
- Experiment II: the feature vector contains left and right DI values
- Experiment III: the feature vector contains all four measures exploited in Experiments I and II
- Experiment IV: the feature vector consists of two items corresponding to MEU and DI of each side merged according to the following formula to obtain a shape-modulated uptake (SMU):

9 $$ \mathrm{S}\mathrm{M}\mathrm{U}=\mathrm{M}\mathrm{E}\mathrm{U}*\left(1-\mathrm{D}\mathrm{I}\right) $$

We trained an additional model using SBR measures obtained by the semi-quantitative approach.

To find out whether the method is useful in decision support, we asked an expert neurologist, blinded to the clinical data, to separate PD patients from control subjects only based on the DaTSCAN qualitative assessment; his classification accuracy was calculated and compared to the other two approaches.

We used the LIBSVM software library [38] to perform all SVM analyses.

### Correlation with motor symptoms

Since it is very important, for any quantitative marker of damage, to reflect the progression of disease symptoms, we explored the relationship between our composite measure SMU and the UPDRS-ME scores from the PD group. We performed a first-correlation analysis without considering the symptom lateralization; we correlated the UPDRS-ME score with the SMU value averaged over both hemispheres. In a second analysis, we split the UPDRS-ME score in the left and right subsections; for each patient, we selected the most affected side (UPDRS-ME-MAS) and correlated it with the contralateral SMU measure (SMU-MAS). Moreover, we investigated the relationship of the SMU measure with the diseases duration and the disease stage using the H&Y rating scale.

We performed all analyses by fitting linear and exponential regression models to the data and calculating the Spearman rank correlation coefficient.

## Results

**Fig. 2:**
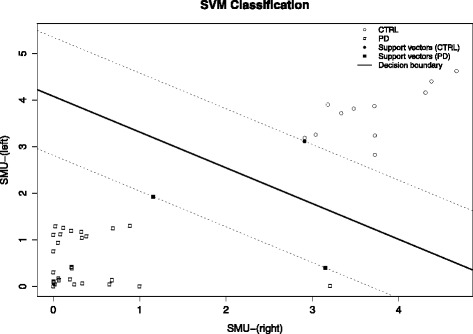
SVM Classiefier results (Experiment 4): scatter plot and hiperplane

We tested the proposed methodology on 43 different SPECT images. We considered segmentation of the basal ganglia to be successful in all cases after visual inspection of the ROIs. We performed ellipsoid fitting on the masks without errors in all subjects. Table 2 shows the classification rates for each SVM model. Figure 2 shows the SVM outcome from Experiment IV. We used right SMU values on the *x*-axis and left SMU values on the *y*-axis to plot the subjects.

**Table 2:** Classification accuracy of the different models

	Exp1 (%)	Exp2 (%)	Exp3 (%)	Exp4 (%)
Correct rate	95.35	0.9767	100	100
AUC	96.77	0.9839	100	100
Specificity	93.55	0.9677	100	100
Sensitivity	100	100	100	100

*AUC* area under curve

**Fig. 3:**
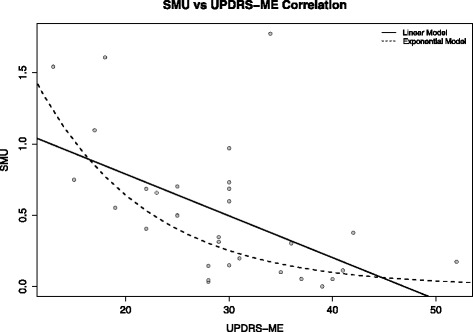
Linear and exponential regression models. SMU (averaged over both hemispheres) versus UPDRS-ME score

Table 3 shows a comparison between our method and more traditional procedures. The proposed methodology outperformed both visual and semi-quantitative assessments that, respectively, achieved accuracies of 97 % (sensitivity 96 %, specificity 100 %) and 95 % (sensitivity 100 %, specificity 83 %). The quantification values obtained by our method showed a good correlation with the UPDRS-ME score (off medication). SMU, averaged over both sides, showed significant negative correlation with the clinical variable (*r*_s_ = −0.55, *p* < 0.01). We observed a comparable relationship in the correlation analyses between the UPDRS-ME-MAS scores and our combined measures. SMU-MAS showed a significant negative correlation with the motor score (*r*_s_ = −0.51, *p* < 0.005). Figure 3 shows both the linear and exponential fits on the scatter plot of our composite measure (SMU) versus the UPDRS motor score. The linear model (*R*^2^_adj_ = 0.26, *p* < 0.01) showed a slightly better fit than the exponential one (*R*^2^_adj_ = 0.22, *p* < 0.01), but both results had the same statistical significance.

**Table 3:** Accuracy comparison between CADA, visual assessment and semi-quantitative assessment

Assessment method	Sensitivity (%)	Specificity (%)	Accuracy (%)
Visual^a^	96	100	97
Semi-quantitative^b^	100	83	95
CADA	100	100	100

^a^Performed by an expert neurologist with years of experience in the field of movement disorders

^b^Performed by nuclear medicine experts blinded to clinical data

No significant correlation was neither found between SMU and the H&Y rating scale, nor between SMU and disease duration.

## Discussion

In this work, we present a novel methodology to process DaTSCAN images. Our aim is not only to obtain a fully automated method able to replicate all essential steps of DaTSCAN analysis, but also to extract a quantitative measure coherently associated with the severity of the symptoms. The proposed method enables the extraction of reproducible measures using the whole SPECT volume and directly connects to striatum uptake and shape. The automated process eliminates the issue of intra- or inter-observer variability. No other imaging data, such as MRI or computed tomography (CT), is needed to perform the analyses. The best results are obtained by providing the classifier both extracted measures, MEU and DI, either merged in a single value or not. Both configurations are able to split subjects without classification errors. Experiments I and II also show high accuracy; this suggests that individual parameters are quite robust. However, best performance exploiting all parameters together suggests the need to provide the classifier with not only intensity/uptake data, but also shape and symmetry features.

Existing semi-quantitative measures of striatal uptake have shown good correlation with UPDRS-ME scores [28, 29]. Moreover, this result seems quite robust, even in the presence of confounding factors such as the assumption of PD medications. Despite the importance of such a relationship in the context of finding a robust marker of disease progression, most studies describing automated DaTSCAN analysis methods do not address the correlation between quantitative measures and clinical scales. Two studies, [20] and [21], are exceptions; the authors reported significant linear relationships between uptake measurements (obtained with BasGan software and Neurostat software, respectively) and the UPDRS-ME score. Our data confirm the association between DaT uptake in striata and motor symptoms, which is consistent with the hypothesis that motor symptoms in PD are related to dopaminergic nigrostriatal pathology [39]. In addition, we also found that a more accurate description of such relationship could be described through an exponential trend, suggesting some sort of saturation effect when the striatum reaches a minimum uptake/dysmorphism value while motor disability continues its progression. This effect could be explained in the advanced stages of PD where the disability progression is related to the severity and extent of extra-nigral degeneration. Moreover, while previous studies employed semi-quantitative measures of mean uptake alone, we were able to confirm the existence of a link between motor symptom severity and a composite index, integrating structural (shape) and functional (uptake) information. Overall, this result suggests that multimodal information extracted from DaTSCAN images may provide further insight into the pathophysiology of parkinsonian syndromes.

Unlike what emerged for the UPDRS-ME, neither of our correlation analyses revealed that neither stage nor disease duration are significantly related to our striatum SPECT measures. A possible explanation of this finding could be the sensitivity of the proposed index to the striatum shape alteration. In fact, the index tends to show a low value, even at the first signs of injury in the putamen, and then quickly levels off. This is consistent with the usual DaT concentration reduction rate, which is faster in the early stages of the disease than in the later stages [28] but can prevent the index from correctly following the disease progression as measured by other clinical features. Moreover, the cross-sectional design and then the spread of data caused by inter-individual differences may have reduced the effectiveness of the correlation. A longitudinal study would better clarify this aspect.

The diagnostic process of PD is not the only scenario that would benefit from the use of reliable, semi-quantitative measures of striatal function, shape and asymmetry, such as those we extracted from DaTSCAN. In fact, our tool could provide a benefit in the diagnostic workup of parkinsonian syndromes and help ameliorate a differential diagnosis from PD in the early stages of the disease or monitor neuroprotective treatments.

It is worth noting that the selected features (MEU and DI) are optimized to the specific clinical setting, i.e. the diagnosis of parkinsonian syndromes. Generally, in patients with PD, the disease progression follows a common pattern; DaT concentration starts to decrease in the putamen contralateral to the clinical symptoms and then progresses to the caudate. When this happens, the striatum loses its characteristic comma-shape on DaTSCAN and tends to become dot-shaped or disappears altogether. In cases of low binding, “forcing” a pre-defined ROI into a comma-shape may bias a semi-quantitative measure, resulting in a SBR that still reaches high values, but that reflects caudate binding only, thus not taking into account putaminal dysfunction. Instead, in our method, striatum changes due to decreased DaT density are observed from different points of view; MEU takes into account uptake changes, while DI detects dysmorphism in the ROI upon which this value is calculated. For what concerns nuclei asymmetry, we did not directly provide any asymmetry measures to any SVM model; we intrinsically introduced asymmetry information into the model since we calculated each parameter on both sides. Both MEU and DI features can be used to derive asymmetry measures.

Although our method has proven to be reliable, we should mention a limitation. To test the method on well-established data, we selected a PD group with a relatively advanced stage of disease; this does not allow clear evaluation of the accuracy of the proposed method in the classification of earlier stages of the disease. As a future work, we intend to select a group of de novo patients that we will follow longitudinally to test this aspect on those with a confirmed PD diagnosis.

## Conclusions

We describe a novel computer-aided diagnosis method for DaTSCAN images. Regions of high uptake are automatically identified from three-dimensional images. The high accuracy obtained by the SVM classifier suggests that the segmentation routine is robust and that the selected parameters well describe the striatum in terms of uptake values, shape and symmetry. Moreover, our measures show high correlation with UPDRS motor score. The comparison with standard qualitative and semi-quantitative techniques suggests that a system based on this method can provide reliable support to the diagnostic process.

## Ethics approval and consent to participate

### Ethical approval

All procedures performed in studies involving human participants were in accordance with the ethical standards of the institutional and/or national research committee and with the 1964 Helsinki declaration and its later amendments or comparable ethical standards.

### Consent to participate

Informed consent to participate was obtained from all individual participants included in the study.

## Consent for publication

Informed consent to publish was obtained from all individual participants included in the study.

## Additional file

Additional file 1: Dysmorphic Index calculation by comparing ellipsoids orientation. (PNG 107 kb)

## Abbreviations

BRASS: Brain Registration and Analysis Software Suite
CT: computed tomography
DaT: dopamine transporter
DI: dysmorphic index
ET: essential tremor
GMM: Gaussian mixture model
H&Y: Hoehn and Yahr (rating scale)
MEU: mean ellipsoid uptake
MRI: magnetic resonance image
PD: Parkinson’s disease
ROI: region of interest
SBR: specific binding ratio
SMU: shape-modulated uptake
SPECT: single-photon emission computed tomography
SVM: support vector machine
UPDRS: Unified Parkinson’s Disease Rating Scale
UPDRS-ME: Unified Parkinson’s Disease Rating Scale Motor Examination

## References

[1] Jankovic J (2008). Parkinson’s disease: clinical features and diagnosis. J Neurol Neurosurg Psychiatry.

[2] Bernheimer H, Birkmayer W, Hornykiewicz O, Jellinger K, Seitelberger F (1973). Brain dopamine and the syndromes of Parkinson and Huntington clinical, morphological and neurochemical correlations. J Neurol Sci.

[3] Kaufman MJ, Madras BK (1991). Severe depletion of cocaine recognition sites associated with the dopamine transporter in Parkinson’s-diseased striatum. Synapse.

[4] Innis RB, Seibyl JP, Scanley BE, Laruelle M, Abi-Dargham A, Wallace E (1993). Single photon emission computed tomographic imaging demonstrates loss of striatal dopamine transporters in Parkinson disease. Proc Natl Acad Sci.

[5] Bhidayasiri R (2006). How useful is (123I) beta-CIT SPECT in the diagnosis of Parkinson’s disease?. Rev Neurol Dis.

[6] Marshall V, Grosset D (2003). Role of dopamine transporter imaging in routine clinical practice. Mov Disord.

[7] Kung H, Kim HJ, Kung MP, Meegalla S, Plössl K, Lee HK (1996). Imaging of dopamine transporters in humans with technetium-99 m TRODAT 1. Eur J Nucl Med.

[8] Koch W, Hamann C, Radau P, Tatsch K (2007). Does combined imaging of the pre- and postsynaptic dopaminergic system increase the diagnostic accuracy in the differential diagnosis of parkinsonism?. Eur J Nucl Med Mol Imaging.

[9] Djang DSW, Janssen MJR, Bohnen N, Booij J, Henderson TA, Herholz K (2012). SNM practice guideline for dopamine transporter imaging with 123I-Ioflupane SPECT 1.0.. J Nucl Med.

[10] Darcourt J, Booij J, Tatsch K, Varrone A, Vander Borght T, Kapucu OL (2010). EANM procedure guidelines for brain neurotransmission SPECT using (123)I-labelled dopamine transporter ligands, version 2. Eur J Nucl Med Mol Imaging.

[11] Wang L, Zhang Q, Li H, Zhang H (2012). SPECT Molecular Imaging in Parkinson’s Disease. J Biomed Biotechnol..

[12] Skanjeti A, Castellano G, Elia BO, Zotta M, Dazzara F, Manfredi M (2015). Multicenter semiquantitative evaluation of (123) I-FP-CIT brain SPECT. J Neuroimaging.

[13] Asenbaum S, Pirker W, Angelberger P, Bencsits G, Pruckmayer M, Brücke T (1998). [123I]β -CIT and SPECT in essential tremor and Parkinson’s disease. J Neural Transm.

[14] Benamer HTS, Patterson J, Grosset DG, Booij J, de Bruin K, van Royen E (2000). Accurate differentiation of parkinsonism and essential tremor using visual assessment of [123I]-FP-CIT SPECT imaging: The [123I]-FP-CIT study group. Mov Disord.

[15] Gerasimou G, Tsolaki M, Bostanjopoulou S, Liaros G, Papanastasiou E, Balaris V (2005). Findings from molecular imaging with SPET camera and 123I-ioflupane in the differential diagnosis of Parkinsonism and essential tremor. Hell J Nucl Med.

[16] Kupsch AR, Bajaj N, Weiland F, Tartaglione A, Klutmann S, Buitendyk M (2012). Impact of DaTscan SPECT imaging on clinical management, diagnosis, confidence of diagnosis, quality of life, health resource use and safety in patients with clinically uncertain parkinsonian syndromes: a prospective 1-year follow-up of an open-label controlled study. J Neurol Neurosurg Psychiatry.

[17] Badiavas K, Molyvda E, Iakovou I, Tsolaki M, Psarrakos K, Karatzas N (2011). SPECT imaging evaluation in movement disorders: far beyond visual assessment. Eur J Nucl Med Mol Imaging.

[18] Gallego J, Ninerola-Baizán A, Cot A, Aguiar P, Crespo C, Falcón C (2015). Validation of semi-quantitative methods for DAT SPECT: influence of anatomical variability and partial volume effect. Phys Med Biol.

[19] Tossici-Bolt L, Hoffmann SA, Kemp P, Mehta R, Fleming J (2006). Quantification of [123I]FP-CIT SPECT brain images: an accurate technique for measurement of the specific binding ratio. Eur J Nucl Med Mol Imaging.

[20] Calvini P, Rodriguez G, Inguglia F, Mignone A, Guerra U, Nobili F (2007). The basal ganglia matching tools package for striatal uptake semi-quantification: description and validation. Eur J Nucl Med Mol Imaging.

[21] Takada S, Yoshimura M, Shindo H, Saito K, Koizumi K, Utsumi H (2006). New semiquantitative assessment of123I-FP-CIT by an anatomical standardization method. Ann Nucl Med.

[22] Koch W, Radau PE, Hamann C, Tatsch K (2005). Clinical testing of an optimized software solution for an automated, observer-independent evaluation of dopamine transporter SPECT studies. J Nucl Med.

[23] Ziebell M, Pinborg LH, Thomsen G, de Nijs R, Svarer C, Wagner A (2010). MRI-guided region-of-interest delineation is comparable to manual delineation in dopamine transporter SPECT quantification in patients: a reproducibility study. J. Nucl. Med. Technol..

[24] Mirzaei S, Zakavi R, Rodrigues M, Schwarzgruber T, Brücke T, Bakala J (2010). Fully automated 3D basal ganglia activity measurement in dopamine transporter scintigraphy (Spectalyzer). Ann Nucl Med.

[25] Illán IA, Górriz JM, Ramírez J, Lang EW, Salas-Gonzalez D, Puntonet CG (2012). Bilateral symmetry aspects in computer-aided Alzheimer’s disease diagnosis by single-photon emission-computed tomography imaging. Artif Intell Med.

[26] Segovia F, Gorriz JM, Ramirez J, Alvarez I, Jimenez-Hoyuela JM, Ortega SJ (2012). Improved parkinsonism diagnosis using a partial least squares based approach. Med Phys.

[27] Martinez-Murcia FJ, Górriz JM, Ramírez J, Moreno-Caballero M, Gómez-Río M (2014). Parametrization of textural patterns in 123I-ioflupane imaging for the automatic detection of parkinsonism. Med. Phys..

[28] Benamer HT, Patterson J, Wyper DJ, Hadley DM, Macphee GJ, Grosset DG (2000). Correlation of Parkinson’s disease severity and duration with 123I-FP-CIT SPECT striatal uptake. Mov. Disord..

[29] Pirker W (2003). Correlation of dopamine transporter imaging with parkinsonian motor handicap: how close is it?. Mov. Disord..

[30] Hughes AJ, Daniel SE, Kilford L (1992). Lees aJ. Accuracy of clinical diagnosis of idiopathic Parkinson’s disease: a clinico-pathological study of 100 cases. J Neurol Neurosurg Psychiatry.

[31] Goetz CG, Fahn S, Martinez-Martin P, Poewe W, Sampaio C, Stebbins GT (2007). Movement Disorder Society-sponsored revision of the Unified Parkinson’s Disease Rating Scale (MDS-UPDRS): process, format, and clinimetric testing plan. Mov Disord.

[32] Goetz CG, Poewe W, Rascol O, Sampaio C, Stebbins GT, Counsell C (2004). Movement Disorder Society Task Force report on the Hoehn and Yahr staging scale: status and recommendations. The Movement Disorder Society Task Force on rating scales for Parkinson’s disease. Mov Disord.

[33] Jenkinson M, Bannister P, Brady M, Smith S (2002). Improved optimization for the robust and accurate linear registration and motion correction of brain images. Neuroimage.

[34] Jenkinson M, Smith S (2001). A global optimisation method for robust affine registration of brain images. Med Image Anal.

[35] Salas-Gonzalez D, Górriz JM, Ramírez J (2013). Illán Ia, Lang EW. Linear intensity normalization of FP-CIT SPECT brain images using the α-stable distribution. NeuroImage.

[36] Balafar MA (2014). Gaussian mixture model based segmentation methods for brain MRI images. Artif. Intell. Rev..

[37] Hartley HO (1958). Maximum likelihood estimation from incomplete data. Biometrics.

[38] Chang CC, Lin CJ. LIBSVM: A library for support vector machines. ACM Trans. Intell. Syst. Technol. 2011;2:27:1–27:27.

[39] Piggott MA, Marshall EF, Thomas N (1999). Striatal dopaminergic markers in dementia with Lewy bodies, Alzheimer’s and Parkinson’s diseases: rostrocaudal distribution. Brain.

