# Supplementary figures and images for: CADA—computer-aided DaTSCAN analysis
Source: EJNMMI Phys. 2016 Feb 16;3:4. doi: 10.1186/s40658-016-0140-9 (PMC4754234; doi:10.1186/s40658-016-0140-9)

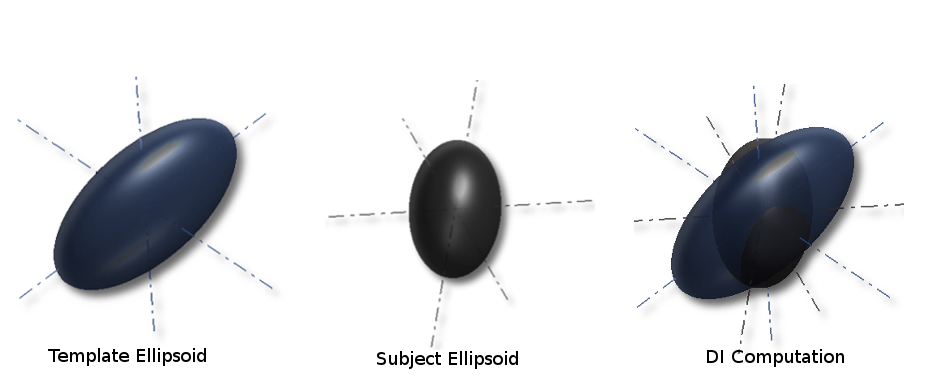

Supplement: Additional file 1: Dysmorphic Index calculation by comparing ellipsoids orientation. — (PNG 107 kb) [file 40658_2016_140_MOESM1_ESM.png]
